# Comparative study of clinical and epidemiological characteristics of major pediatric adenovirus epidemics in southern Taiwan

**DOI:** 10.1186/s12879-019-4305-8

**Published:** 2019-08-01

**Authors:** Ching-Fen Shen, Shih-Min Wang, Jen-Ren Wang, Yu-Shiang Hu, Tzong-Shiann Ho, Ching-Chuan Liu

**Affiliations:** 1 Department of Pediatrics, National Cheng Kung University Hospital, College of Medicine, National Cheng Kung University, 138, Sheng Li Road, North Dist, Tainan, 70403 Taiwan; 2 Department of Emergency Medicine, National Cheng Kung University Hospital, College of Medicine, National Cheng Kung University, 138, Sheng Li Road, North Dist, Tainan, 70403 Taiwan; 3 Department of Medical Laboratory Science and Biotechnology, College of Medicine, National Cheng Kung University, 138, Sheng Li Road, North Dist, Tainan, 70403 Taiwan; 4 Department of Pathology, National Cheng Kung University Hospital, College of Medicine, National Cheng Kung University, 138, Sheng Li Road, North Dist, Tainan, 70403 Taiwan; 5 Center of Infectious Disease and Signaling Research, National Cheng Kung University Hospital, College of Medicine, National Cheng Kung University, 138, Sheng Li Road, North Dist, Tainan, 70403 Taiwan

**Keywords:** Adenovirus, Genotype, Epidemic, Pneumonia, Hospitalization, Risk factors

## Abstract

**Background:**

Human adenoviruses (HAdV) are important pathogens of pediatric respiratory tract infections in Taiwan. There were two major HAdV epidemics in southern Taiwan in 2011 and 2014, respectively.

**Methods:**

The demographic, clinical characteristics, and risk factors for hospitalization of pediatric patients with HAdV infection in the two outbreaks were retrospectively compared. The epidemic was defined as > 7% HAdV detection rate for six consecutive weeks. HAdV infection was defined as positive HAdV isolates from respiratory tract specimens. HAdV genotype was determined by PCR-based hexon gene sequencing.

**Results:**

A total of 1145 pediatric patients were identified (635 cases in 2011; 510 cases in 2014). HAdV genotype 3 and 7 contributed to both epidemics, although the proportion of HAdV3 decreased significantly (64.7% in 2011 to 25.5% in 2014, *p* < 0.001) and was replaced by other genotypes (type 1, 4, and 6) in the 2014 epidemic. Among the hospitalized patients, there were more patients hospitalized with bronchopneumonia/or pneumonia in the 2011 epidemic (10.6% vs 5.1%, *p* < 0.001), while more patients hospitalized with acute pharyngitis/pharyngoconjunctival fever (63.9% vs. 38.6%, *p* < 0.001) in the 2014 epidemic. In both epidemics, hospitalized patients had higher WBC and C-reactive protein (CRP) levels than non-hospitalized patients. Using multivariate regression analysis, underlying disease and elevated CRP levels were independent risk factors for hospitalization in both epidemics.

**Conclusion:**

There were significant differences in clinical, viral characteristics and risk factors of hospitalization between the 2011 and 2014 epidemics. Understanding changes in the epidemiological and clinical characteristics of HAdV epidemics is important from a public health perspective.

## Highlights

- The 2011 and 2014 HAdV epidemics in Taiwan were caused by different HAdV genotypes.
- Hospitalized patients had higher WBC and CRP levels than non-hospitalized patients.
- Underlying disease and elevated CRP were independent risk factors of hospitalization.

## Background

Human adenoviruses (HAdVs) are double stranded non-enveloped DNA viruses which cause a wide range of illnesses including acute respiratory infections (ARIs), gastroenteritis, conjunctivitis, cystitis, and meningoencephalitis. HAdV infection accounts for 5–7% of respiratory illnesses in children and infants [1, 2]. HAdV-associated acute lower respiratory tract infections (ALRTIs) may be severe and lead to long term respiratory sequelae, such as bronchiolitis obliterans or bronchiectasis [3]. HAdVs were an important cause of acute respiratory tract infections in Taiwan [4]. Studies have shown that the most susceptible populations were children < 5 years old, close-quartered populations such as schools and military training camps, and immunocompromised individuals [5]. Currently, there are more than 60 serotypes of HAdVs, classified into 7 species (A-G). Molecular typing of HAdVs is based on PCR-based direct sequencing of the HAdV hexon gene which contains 7 hypervariable regions [6]. Different serotypes were associated with different clinical manifestations and various degrees of disease severity [7, 8]. Serotypes 3, 4, 7 and 21 have been reported to be associated with severe disease, and HAdV7 was related to post-infectious pulmonary sequelae, such as bronchiolitis obliterans [9–11]. Molecular genotyping of HAdV was used to analyze the dynamic changes in epidemiology and the association with disease manifestations [7, 8].

There was a significant increase in the incidence of HAdV infection in southern Taiwan between 1999 and 2002, with three outbreaks of adenovirus respiratory infection between November 1999 and December 2001. HAdV3 and HAdV7 were the major serotypes in the first outbreak, while HAdV4 was the major serotype in the second and third outbreak [7, 12, 13]. Species B, especially HAdV3 has been reported to be the predominant respiratory HAdV in Taiwan over the past decade, and has been identified in half of all children hospitalized with respiratory infections [14]. Other outbreaks recorded by the nationwide surveillance system included an outbreak in 2004–2005 which was predominantly caused by HAdV3 [7, 15, 16], and an outbreak which occurred between March and October 2011 by HAdV3 and HAdV7 [17, 18].

The most recent HAdV outbreak in Taiwan was in 2014. However, currently available molecular epidemiology data from this outbreak have not been completely analyzed. In this study, we conducted a molecular characterization of HAdV from the 2014 epidemic, and compared the demographics, clinical characteristics, and risk factors of hospitalization and pneumonia in patients between the 2011 and 2014 epidemics.

## Methods

### Study design

This study retrospectively enrolled pediatric patients with virologically-confirmed HAdV infection who presented at the National Cheng Kung University Hospital (NCKUH), a national university-affiliated, 1200 bed teaching hospital, which is also the major tertiary referral center in southern Taiwan. This study enrolled patients (< 18 years old) who were diagnosed with HAdV between Nov. 2010 to Dec. 2015. HAdV infection is defined based on the presence of HAdV isolated from clinical specimens, including nasopharyngeal aspirates, throat or nasal swabs. The patient list of HAdV infections was abstracted from the hospital-based administrative electronic data. Patients without complete clinical information available for analysis and adult patients were excluded. The NCKUH virology laboratory is one of the contracted laboratories of the nationwide viral surveillance system of the Center for Disease Control (CDC), Taiwan. HAdV epidemics are defined as HAdV-positive rate above 7.0% for six consecutive weeks according to baseline epidemiological surveys [19] . The chest X-rays were independently interpreted by qualified radiologists who were blinded to the clinical information. This study was approved by the Institutional Review Board (IRB) of the NCKUH (No. ER-100-060).

### Viral culture and genotyping

Throat swabs, nasopharyngeal swabs or aspirates were collected in viral transport medium, and inoculated into tubes containing Madin-Darby canine kidney cells, Vero cells, A549 cells or rhabdomyosarcoma cells within 24 h of collection. Cells were incubated at 37 °C and viral cytopathic effect was evaluated daily for a minimum of 14 days. HAdV infection was confirmed with immunofluorescent assays using D^3^ Ultra DFA (direct fluorescent antibody) respiratory virus screening and identification kit (Diagnostic Hybrids, Inc., Athens, OH, USA). After immunofluorescent confirmation of HAdV infection, the cell culture lysate were stored at − 70 °C for further genotype determination. Viral DNA was extracted from 200 μL of the original adenovirus-positive specimen using the QIAamp DNA Blood Mini Kit (Qiagen) according to the manufacturer’s instructions. A 1004 bp fragment of the hexon gene was PCR-amplified from 5 μL of eluted samples using primers AdTU7 (5′-GCCACCTTCTTCCCCATGGC-3′) and AdTU4′ (5′-GTAGCGTTGCCGGCCGAGAA-3′). These amplicons were then sequenced on a 3730 xl DNA Analyzer (Applied BioSystems). The forward and reverse sequences were combined using BioEdit (Ibis Therapeutics) and sequence alignment was performed with 51 previously characterized prototype viruses, as well as other characterized genotypes from GenBank and our internal sequence collection. A good genotype matche was defined as specimens which resulted in > 90% alignment identify score.

### Statistical analysis

Categorical variables were presented as counts and percentages and tested by Chi-square test or Fisher’s exact test. Continuous variables were presented as mean and standard deviation and tested by independent sample t-test. Variables with *p* < 0.05 through univariate logistic regression analysis were selected into the multivariate logistic regression model. Multivariate logistic regression analysis was performed to investigate the risk factors associated with hospitalization and pneumonia. All statistical analyses were performed using the IBM SPSS statistical software version 22 for Windows (IBM Corp., Armonk, New York, USA). Two-tailed *P* values < 0.05 were considered statistically significant.

## Results

### Epidemiology of HAdV infection during 2010–2015

**Fig. 1:**
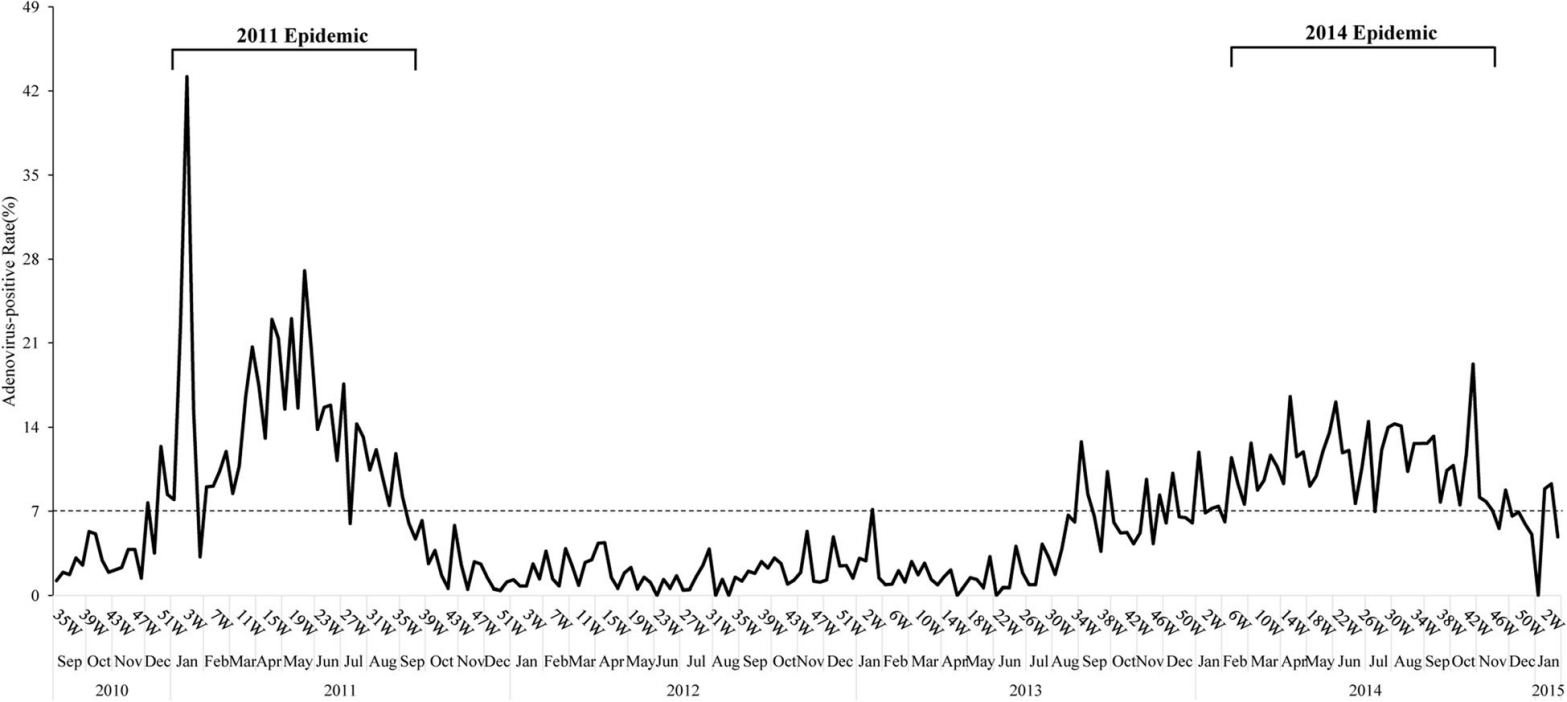
HAdV-positive rates between 2010 to 2015 at the NCKU hospital in southern Taiwan. The monthly distribution of HAdV infection, the peaks during the two epidemics, and the reference line of the threshold rate are all represented

This study reviewed the medical records of 1717 virologically-confirmed HAdV-infected patients. In brief, all enrolled patient should have complete physical examination record and virological workup. All cases in this cohort had HAdV isolated from viral culture from respiratory samples, including (throat swab, nasal swab, or nasopharyngeal aspirates); cases with prolonged fever or severe enough to visit ER, or warranting hospitalization received further investigation, including urine analysis, blood culture, or bacterial culture from sterile or non-sterile sites. Blood culture results were evaluated for all febrile hospitalized children in order to exclude occult bacteremia. Isolation of other respiratory pathogens including bacteria, or viruses other than HAdV was defined as co-infection. However, patients with severe definite bacterial infection, including bacteremia and urinary tract infection were excluded from this cohort. Fifty-three adult patients were also excluded. Analysis of the monthly distribution of HAdV infection showed that HAdV circulated year-round, but there were two major HAdV outbreaks during the study period (Fig. 1). The first epidemic comprised 635 patients, and occurred between week 51 of 2010 and week 39 of 2011. The second wave comprised 510 patients, and occurred between week 6 and week 46 of 2014. The HAdV-positive rate was defined as the number of HAdV-positive respiratory specimens/total number of respiratory specimens X100. The baseline HAdV-positive rate was 5.45% ± 4.53% (range 0–26.3%) during 2010–2015 except for the period during the epidemics. The mean HAdV-positive rates during these two epidemics were 36.2% in 2011 and 11.3% in 2014, which were significantly higher than the non-outbreak period (*P* < 0.001) (Fig. 1).

### Comparison of demographics, clinical characteristics and HAdV genotype in patients between 2011 and 2014 epidemics

We compared the demographics and clinical characteristics of patients between the 2011 and 2014 HAdV epidemics (Table 1). There were no significant differences in mean age, gender, or presence of co-infections with bacteria or other viruses between the two epidemics. A significantly higher proportion of patients had acute pharyngitis/pharyngoconjunctival fever (PCF)-related symptoms (fever, infected throat, cough, rhinorrhea and conjunctivitis) in the 2014 epidemic compared to the 2011 epidemic (all *p* < 0.05), while a significantly higher proportion of patients had acute gastroenterocolitis-related symptoms (diarrhea and vomiting) in the 2011 epidemic (all *p* < 0.05). The 2011 epidemic had a significantly higher percentage of genotype 2 and genotype 3 infections compared to the 2014 epidemic (both *p* < 0.001), while the 2014 epidemic had a significantly higher percentage of genotype 1, genotype 4 and genotype 6 infections (all *p* < 0.001).

**Table 1:** Comparison of demographics, clinical characteristics and genotypes of patients with HAdV infection between 2011 and 2014 epidemics

Variables	2011 epidemics (*N* = 635)	2014 epidemics (*N* = 510)	*P*-value
Age (years)	4.8 ± 3.3	4.9 ± 3.4	0.581
Gender (M/F ratio)	1.19	1.42	0.145
Co-infection	15 (2.4)	22 (4.3)	0.063
Underlying disease	57 (9.0)	31 (6.1)	0.067
Diagnosis			
Upper respiratory			
Acute pharyngitis/ PCF	423 (66.6)	385 (75.5)	0.001*
Lower respiratory			
Bronchiolitis /bronchopneumonia	134 (21.1)	113 (22.2)	0.666
Pneumonia	21 (3.3)	8 (1.6)	0.063
Acute gastroenterocolitis	60 (9.4)	30 (5.9)	0.026*
Clinical Manifestation			
Fever	546 (86.0)	510 (100.0)	< 0.001*
Infected throat	563 (88.7)	496 (97.3)	< 0.001*
Cough	410 (64.6)	422 (82.7)	< 0.001*
Rhinorrhea	341 (53.7)	403 (79.0)	< 0.001*
Conjunctivitis	51 (8.0)	69 (13.5)	0.003*
Abdominal pain	99 (15.6)	89 (17.5)	0.398
Diarrhea	153 (24.1)	87 (17.1)	0.004*
Vomiting	218 (34.3)	136 (26.7)	0.005*
Survival rates	633 (99.7)	510 (100.0)	0.506
Genotype (total number)	*n* = 156	*n* = 157	
Type1	8 (5.1)	44 (28.0)	< 0.001*
Type2	18 (11.5)	1 (0.6)	< 0.001*
Type3	101 (64.7)	40 (25.5)	< 0.001*
Type4	0 (0)	25 (15.9)	< 0.001*
Type5	2 (1.3)	0 (0)	0.248
Type6	1 (0.6)	14 (8.9)	< 0.001*
Type7	26 (16.7)	33 (21.0)	0.325

Note: M/F ratio, male to female ratio; PCF, pharyngoconjunctival fever

* *P* < 0.05

### Comparison of hospitalized and non-hospitalized patients between the 2011 and 2014 epidemics

Differences in demographics and clinical characteristics of hospitalized and non-hospitalized patients were compared between the 2011 and 2014 epidemic (Table 2). In the 2011 epidemic, the mean age of non-hospitalized patients was significantly higher compared to hospitalized patients (5.0 vs. 4.4 years, *p* = 0.045). There was a significantly higher percentage of non-hospitalized patients with acute pharyngitis/PCF (*p* < 0.001), while there was a significantly higher percentage of bronchiolitis/bronchopneumonia (*p* < 0.001), pneumonia (*p* < 0.001) and acute gastroenterocolitis (*p* = 0.016) among hospitalized patients. A significantly higher percentage of hospitalized patients had underlying disease compared to non-hospitalized patients (*p* = 0.001). Clinical manifestations showed that a significantly higher percentage of non-hospitalized patients had injected throat (*p* = 0.004) compared with hospitalized patients, while a significantly higher percentage of hospitalized patients had cough and diarrhea compared with non-hospitalized patients (both *p* < 0.05). Laboratory values showed that hospitalized patients had significantly higher mean WBC (12,044 vs. 5051 cells/μL, *p* < 0.001), absolute lymphocyte count (ALC) counts (2624 vs. 2138 cells/μL, *p* = 0.015), and mean CRP values (80.8 vs. 47.6 mg/L, *p* < 0.001) compared to non-hospitalized patients.

**Table 2:** Comparison of demographics and clinical characteristics of hospitalized and non-hospitalized patients of HAdV infection between 2011 and 2014 epidemics

Variables	2011 epidemics		2014 epidemics		Significance of comparisons		
	Non-hospitalization	Hospitalization	Non-hospitalization	Hospitalization	P value^1^	P value^2^	P value^3^
Age	5.0 ± 3.1	4.4 ± 3.5	5.1 ± 3.3	4.7 ± 3.5	0.045	0.209	0546
Gender (M/F ratio)	1.08	1.57	1.33	1.63	0.072	0.297	0.671
Co-infection	7 (1.6)	8 (4.2)	6 (1.7)	16 (10.1)	0.081	< 0.001*	0.031
Underlying disease	29 (6.5)	28 (14.8)	8 (2.3)	23 (14.6)	0.001*	< 0.001*	0.946
Diagnosis							
Acute pharyngitis/PCF	350 (78.5)	73 (38.6)	284 (80.7)	101 (63.9)	< 0.001	< 0.001	< 0.001
Bronchiolitis/bronchopneumonia	60 (13.5)	74 (39.2)	55 (15.6)	58 (36.7)	< 0.001	< 0.001*	0.640
Pneumonia	1 (0.2)	20 (10.6)	0 (0.0)	8 (5.1)	< 0.001	< 0.001	0.006
Acute gastro-enterocolitis	34 (7.6)	26 (13.8)	16 (4.5)	14 (8.9)	0.016	0.055	0.155
Clinical manifestation							
Fever	378 (84.8)	168 (88.9)	352 (100.0)	158 (100.0)	0.17	–	< 0.001*
Injected throat	406 (91.0)	157 (83.1)	344 (97.7)	152 (96.2)	0.004	0.381	< 0.001*
Cough	259 (58.1)	151 (79.9)	282 (80.1)	140 (88.6)	< 0.001*	0.019	0.028*
Rhinorrhea	229 (51.3)	112 (59.3)	271 (77.0)	132 (83.5)	0.067	0.093	< 0.001*
Conjunctivitis	35 (7.8)	16 (8.5)	46 (13.1)	23 (14.6)	0.793	0.649	0.074
Abdominal pain	66 (14.8)	33 (17.5)	59 (16.8)	30 (19.0)	0.398	0.540	0.713
Diarrhea	97 (21.7)	56 (29.6)	43 (12.2)	44 (27.8)	0.034	< 0.001*	0.715
Vomiting	146 (32.7)	72 (38.1)	75 (21.3)	61 (38.6)	0.193	< 0.001*	0.922
Laboratory monitor							
WBC (cells/μL)	5051 ± 6336	12,044 ± 5983	6000 ± 6683	13,130 ± 5825	< 0.001	< 0.001*	0.089
ANC (cells/μL)	14,857 ± 77,292	8312 ± 5207	15,095 ± 101,895	9076 ± 4865	0.217	0.465	0.163
ALC (cells/μL)	2138 ± 1500	2624 ± 2307	2456 ± 1608	2670 ± 2097	0.015	0.286	0.847
CRP (mg/L)	47.6 ± 33.4	80.8 ± 71.5	52.4 ± 39.5	77.4 ± 63.3	< 0.001*	< 0.001*	0.641
Survival rates	445 (99.8)	188 (99.5)	352 (100.0)	158 (100.0)	–	–	0.360

Note: M/F ratio, male to female ratio; PCF, pharyngoconjunctival fever; WBC, white blood cell counts; ANC, absolute neutrophil counts; ALC, absolute lymphocyte counts; CRP, C-reactive protein

^123^, *p*-value of comparison between non-hospitalization and hospitalization in ^1^2011 epidemics, ^2^2014 epidemics, and *p*-value of ^3^comparison between 2011 and 2014 epidemics given hospitalization

* *P* < 0.05

During the 2014 epidemic, a higher percentage of hospitalized patients had co-infections compared to non-hospitalized patients (*p* < 0.001). There was a significantly higher percentage of non-hospitalized patients with acute pharyngitis/PCF compared to hospitalized patients (*p* < 0.001), while a significantly high percentage of hospitalized patients had bronchiolitis/bronchopneumonia and pneumonia (both *p* < 0.001). A higher percentage of hospitalized patients had underlying disease (*p* < 0.001). There was a significantly higher percentage of non-hospitalized patients with cough and vomiting compared to hospitalized patients (both *p* < 0.05). Laboratory values showed that hospitalized patients had significantly higher mean WBC counts (13,130 vs. 6000 cells/μL, *p* < 0.001), and CRP values (77.4 vs. 52.4 mg/L, *p* < 0.001) compared to non-hospitalized patients.

Comparison of hospitalized patients during the 2011 and 2014 epidemics showed higher percentage of patients had co-infection (10.1% vs. 4.2%, *p* = 0.031), with diagnosis of acute pharyngitis/PCF (63.9% vs. 38.6%, *p* = 0.031), and presented the symptoms of fever (100.0% vs. 88.9%), injected throat (96.2% vs. 83.1%), cough (88.6% vs. 79.9%) and rhinorrhea (83.5% vs. 59.3%, all *p* < 0.05) during the epidemic 2014. However, higher proportion of patients were hospitalized with the diagnosis of pneumonia in the 2011 epidemic than in the 2014 epidemic (10.6% vs. 5.1% *p* = 0.006).

### Risk factors associated with hospitalization in the 2011 and 2014 epidemics

Multivariate logistic regression analysis to identify the risk factors associated with each epidemic was listed in Table 3. In the 2011 epidemic, the risk of hospitalization was significantly increased in patients with underlying disease (OR = 2.747, *p* = 0.015), cough (OR = 1.959, *p* = 0.017), and increased CRP values (OR = 1.015, *p* < 0.001). In the 2014 epidemic, the risk of hospitalization was significantly increased in patients with co-infections (OR = 5.944, *p* = 0.011), bronchiolitis/ bronchopneumonia (OR = 2.505, *p* = 0.024), underlying disease (OR = 18.187, *p* < 0.001), diarrhea (OR = 2.056, *p* = 0.037), vomiting (OR = 2.413, *p* = 0.003), increased WBC counts (OR = 1.00006, *p* = 0.018) and increased CRP values (OR = 1.007, *p* = 0.007). Using a cutoff value of CRP = 60 in univariate logistic regression analysis, in the 2011 and 2014 epidemics, the risk of hospitalization was significantly increased in patients with CRP values > 60 (supplementary Table S1; OR = 2.333 and 2.833, respectively, all *p* < 0.05). Underlying disease and elevated CRP level were independent risk factors for hospitalization in both epidemics.

**Table 3:** Multivariate logistic regression analysis of risk factors associated with hospitalization in the 2011 and 2014 epidemics

Variables	2011 epidemic		2014 epidemic	
	OR (95% CI)	*p*-value	OR (95% CI)	*p*-value
Age (years)	0.961 (0.875, 1.058)	0.425		
Co-infection (yes vs. no)			5.944 (1.504,23.486)	0.011*
Diagnosis				
Acute pharyngitis/ PCF (yes vs. no)	0.673 (0.308, 1.472)	0.321	1.234 (0.564,2.699)	0.598
Bronchiolitis /bronchopneumonia (yes vs. no)	2.278 (0.995, 5.214)	0.051	2.505 (1.128,5.563)	0.024*
Acute gastroenterocolitis (yes vs. no)	1.711 (0.793, 3.690)	0.171		
Underlying disease (yes vs. no)	2.747 (1.214, 6.212)	0.015*	18.187 (4.571,72.360)	< 0.001*
Clinical Manifestation				
Injected throat (yes vs. no)	0.469 (0.195, 1.125)	0.090		
Cough (yes vs. no)	1.959 (1.128, 3.405)	0.017*	1.840 (0.818,4.141)	0.141
Diarrhea (yes vs. no)	1.127 (0.641, 1.980)	0.679	2.056 (1.043,4.050)	0.037*
Vomiting (yes vs. no)			2.413 (1.358,4.288)	0.003*
Laboratory monitor				
WBC (cells/μL)	1.00003 (0.99997, 1.00001)	0.333	1.00006 (1.00001,1.00011)	0.018*
ALC (cells/μL)	1.00005 (0.99984, 1.00026)	0.670		
Seg (%)	0.976 (0.955, 0.998)	0.033*		
Band (%)			1.032 (0.996,1.070)	0.078
CRP (mg/L)	1.015 (1.009,1.021)	< 0.001*	1.007 (1.002,1.013)	0.007*

Note: *PCF* pharyngoconjunctival fever, *WBC* white blood cell counts, *ALC* absolute lymphocyte counts, *CRP* C-reactive protein, *OR* odds ratio; CI, confidence intervals

* *P* < 0.05

### Comparison of difference between patients with and without HAdV pneumonia

Diagnosis of radiologically-conformed alveolar pneumonia was according to World Health Organization (WHO) criteria [20]. Presence of dense opacity could suggest the following: consolidation of a part/entire lobe or whole lung (could contain air bronchograms or could associate with pleural effusion), or pleural effusion associated with pulmonary infiltration, or an large effusion enough to obscure such an opacity”.

There were 29 patients diagnosed with HAdV pneumonia in the two epidemics. We compared the demographics and clinical characteristics of these 29 patients with non-pneumonia patients (Table 4). There were no significant differences in age or gender between these two groups. However, a significantly higher percentage of pneumonia patients had co-infections compared to non-pneumonia patients (*p* = 0.012). Among the co-infections, viral infection (75%) was more predominant than bacterial infection in the pneumonia group. Pneumonia patients had significantly higher mean WBC counts (11,334.8 vs. 7515.1 cells/μL, *p* < 0.001), and CRP levels (98.9 vs. 62.2 mg/L, *p* = 0.046), but lower mean ALC (1697.9 vs. 2484.7 cells/μL, *p* = 0.001) compared to non-pneumonia patients. There was no significant difference in genotype distribution between pneumonia patients and non-pneumonia patients.

**Table 4:** Comparison of demographics and clinical characteristics between the pneumonia and non-pneumonia patients in 2011 and 2014 epidemics

Variables	Non-pneumonia (*N* = 1116)	Pneumonia (*N* = 29)	*p*-value
Age (years)	4.9 ± 3.3	4.9 ± 3.8	0.964
Gender (M/F ratio)	624/492	20/9	0.162
Underlying disease	86 (7.7)	2 (6.9)	0.999
Co-infection	33 (3.0)	4 (13.8)	0.012*
Virus	13 (39.4)	3 (75.0)	
Bacteria	20 (60.6)	1 (25.0)	
Laboratory monitor			
WBC (cells/μL)	7515.1 ± 7139.5	11,334.8 ± 5031.3	< 0.001*
ANC (cells/μL)	12,183.1 ± 67,084.4	9010.5 ± 3856.9	0.803
ALC (cells/μL)	2484.7 ± 1917.5	1697.9 ± 1061.8	0.001*
CRP (mg/L)	62.2 ± 53.1	98.9 ± 92.1	0.046*
Genotype (total number)	*n* = 291	*n* = 22	
type 1	48 (16.5)	4 (18.2)	0.770
type 2	19 (6.5)	0 (0)	0.379
type 3	129 (44.3)	12 (54.5)	0.353
type 4	24 (8.2)	1 (4.5)	0.537
type 5	2 (0.7)	0 (0)	0.999
type 6	14 (4.8)	1 (4.5)	0.999
type 7	55 (18.9)	4 (18.2)	0.999

Note: M/F ratio, male to female ratio; ANC, absolute neutrophil counts; ALC, absolute lymphocyte counts; CRP, C-reactive protein

* *P* < 0.05

## Discussion

In this study, we compared the demographics, clinical characteristics, and risk factors of hospitalization and pneumonia in patients from the 2011 and 2014 adenovirus epidemics in southern Taiwan. Genotype 2, 3 and 7 accounted for the majority of infections in the 2011 epidemic. Although genotype 3 and 7 sustained to contribute to the 2014 epidemic, these genotypes comprised < 50% of cases in the 2014 epidemic. The surge of other genotypes, including 1, 4 and 6 constituted the other half of genotypes found in the 2014 epidemic. In addition to genotype, there were also differences in the clinical characteristics, laboratory values, and hospitalization between the two epidemics. Our analysis showed that the 2011 epidemic was mainly caused by HAdV3 and HAdV7, with a higher prevalence of HAdV3 compared to HAdV7 (64.7% vs. 16.7%). This finding was consistent with other epidemiological reports, showing co-circulation of HAdV3 and HAdV7 in the 2011 epidemic [17–19, 21]. HAdV3 has been the most prevalent genotype in Taiwan since 1980, and also contributed to 2004–2005 epidemic [7, 13, 15]. HAdV3 was also the most prevalent genotype in nearby countries. Lee et al. had reported a pandemic outbreak of acute respiratory infections caused by HAdV3 in 2010 in Korea, which preceded our 2011 epidemic [22]. In China, Gao and colleagues also reported an epidemiology study of HAdV outbreaks among children in Aug 2010 and Jul 2011, in which HAdV3 (70.1%) was the leading genotype, followed by HdAV7 [23]. Previous reports showed that HAdV-7 was rarely detected in Taiwan until 1999–2000. However, by the 1999 outbreak, HAdV-7 had emerged as the predominant genotype (45%), even over the HAdV-3 (36%) [13]. Since then, HAdV-7 has been consistently detectable through epidemiological surveys, but has accounted for a much smaller proportion (ranging from 0.7 to 1.2%) [7, 15]. The re-emergence of HAdV-7 during 2011 epidemic in Taiwan paralleled the re-emergence of HAdV-7 in southern China, suggesting possible viral spreading through a nearby country [23, 24]. Although HAV55 and HAdV11 were also reported to be other important emergent genotypes in immunocompetent adults with severe pneumonia in China, we did not identify any HAdV55 or HAdV11 in this study [25, 26]. Continuous epidemiological surveillance is still needed to monitor dynamic changes of HAdV serotypes.

The clinical presentations varied among different HAdV serotypes. Lin et al. reported that HAdV3 more likely to cause upper respiratory tract infections, while HAdV7 tends to cause lower respiratory tract infections and a more severe clinical course, including longer duration of fever and hospital stay, and need for intensive care [27]. Many other reports also demonstrated that infection with HAdV7 was more likely to be associated pneumonia [11, 17]. On the contrary, we found that the 2014 epidemic had a higher rate of HAdV7 compared to the 2011 epidemic (21.0% vs. 16.7%), but had a lower rate of pneumonia (1.6% vs. 3.3%). Also, when we pooled these two epidemics into analysis, we found that infection with genotype 7 was not statistically associated with pneumonia. HAdV4 caused an epidemic outbreak of acute respiratory disease (ARD) in military recruits in United States and may also result in severe pneumonia [28, 29]. HAdV1 was also reported to cause severe pneumonia in immunocompetent patients in French intensive care unit [30]. Our study showed that although HAdV1 and HAdV4 were two of the emerging genotypes in the 2014 epidemic, they were not associated with severe lower respiratory tract infection (four pneumonia cases caused by HAdV1; one pneumonia case caused by HAdV4). It is therefore possible that there may have some genomic change within the same HAdV genotype, resulting in different invasiveness and virulence. Sub-serotype or phylogenetic analysis is needed for further study of serotype-specific manifestation.

As well as viral genomic characteristics, host susceptibility is a major risk factor for severe HAdV infection. The neurological disease, chronic lung disease, and airway anomalies were reported to be related to HAdV pneumonia [19, 21]. Neurological disease was particularly associated with severe pneumonia caused by HAdV3 and 7 during the 2011 epidemic [17]. Risk factors of hospitalization, including neurologic, respiratory, and metabolic abnormalities, were revealed to be associated with more severe HAdV disease requiring hospitalization [31]. Although we did not sub-analyze the underlying disease by disease categories, underlying disease for HAdV associated hospitalization in both epidemics was demonstrated.

Our data showed that in both epidemics, hospitalized patients had higher WBC counts and CRP levels compared with non-hospitalized patients. Leukocytosis and elevated CRP levels are common in HAdV infection, even without superimposed bacterial infection [8]. However, leukocytosis of HAdV infection could be genotype-specific, and also related to the stage of clinical infection. Lin et al. had reported that leukocytosis (WBC > 15,000/mm3) was more common in HAdV2 infection, whereas leukopenia (WBC < 5000/mm3) was more common in HAdV7 infection [27]. Leukocytosis seems to occur in the early course of infection, whereas leukopenia and thrombocytopenia were shown to correlate with progression [17, 32]. This could also explain some reports showing leukocytosis as the major laboratory finding in HAdV infection, while other studies reported leukopenia [17, 19, 21]. In our study, we demonstrated that elevation of CRP values was an independent predictor of hospitalization in both epidemics. Previous data suggested that serum CRP levels were a better predictor of bacterial infection in febrile children compared to WBC, ANC or ABC counts [33]. Elevated CRP levels in children with HAdV infection in the absence of secondary bacterial infection indicated that HAdV triggered an inflammatory host response similar to that of a bacterial infection [34]. However, the elevation of CRP could be serotype-specific, since children infected with HAdV3 had higher CRP values compared to children infected with other genotypes [15].

HAdV pneumonia is the most common manifestation of severe HAdV infection in immunocompetent patients, but accounts for less 5% of all HAdV infection [35]. In our analysis, pneumonia patients had higher WBC counts and CRP levels than non-pneumonia patients. This was consistent with a previous study, which showed that leukocytosis was seen in one-fourth of the patients and a moderately elevated CRP > 40 mg/L was seen in nearly two-thirds of the patients [35].

As discussed by Chen et al. study [36], seven species of adenovirus (A–G), including more than 79 genotypes, have been defined using a new characteristic based on genomics [37]. Specific genotypes are often associated with particular clinical manifestations [38, 39]. HAdV species B contains several types related to acute respiratory diseases (ARD), can be further classified into two subtypes B1 and B2 based on their tissue tropism. HAdV types 3 and 7 of species B are most commonly detected in patients with respiratory infections [40, 41]. Since 2006, there are the outbreaks and severe respiratory disease worldwide, which were caused by HAdV14p1 and HAdV55 of species B [42, 43]. These two strains have the potential to extensively disseminate and cause severe epidemics due to the lack of herd immunity and their ability to cause more severe ARD than other adenoviruses [44]. In the present study, we showed that the 2011 and 2014 HAdV epidemics in Taiwan were caused by different HAdV genotypes. In addition, hospitalized patients had higher WBC and CRP levels than non-hospitalized patients. There is a need for a licensed HAdV vaccine for the general population, as the only approved live oral vaccine (comprising HAdV types 4 and 7) has only been used in the US military [45, 46].

There are some limitations in this study. First, molecular typing was done in only 24 and 30% of all HAdV isolates, in 2011 and 2014 respectively through random sampling. But, the number of viral isolates chosen for further genotype in each epidemic is relative large compared with all other studies conducted in Taiwan during the same period of time. Also, our genotype distribution result is compatible with other epidemiological studies in Taiwan in 2011 [19]. Second, in our study, we only enrolled cases with HAdV isolated from clinical culture. Although viral culture had been replaced by nucleic acid detection using multiplex PCR in many developed countries, it is more expensive and not yet covered by Taiwan’s health insurance system.

## Conclusion

We found co-circulation and emergence of different HAdV genotypes in the 2011 and 2014 epidemics. The 2011 epidemic was mainly caused by HAdV genotype 3 and 7 and had more gastrointestinal symptoms, while the 2014 epidemic was caused by genotype 1, 2, 3, and 7 and had more upper respiratory tract infections. Underlying disease and high CRP levels were independent risk factors of hospitalization in both epidemics. Understanding the changes in epidemiological and clinical characteristics of HAdV epidemics is important from a public health perspective.

## Data Availability

The data used to support the findings of this study are included within the article.

## Abbreviations

ABC: Absolute band count
ALRTIs: Acute lower respiratory tract infections
ANC: Absolute neutrophil count
ARD: Acute respiratory disease
ARIs: Acute respiratory infections
CRP: C-Reactive protein
HAdV: Human adenoviruses
PCF: Pharyngoconjunctival fever
WBC: White blood cell
